# PR3-ANCA and panel diagnostics in pediatric inflammatory bowel disease to distinguish ulcerative colitis from Crohn's disease

**DOI:** 10.1371/journal.pone.0208974

**Published:** 2018-12-17

**Authors:** Michael P. Horn, Anna Maria Peter, Franziska Righini Grunder, Alexander B. Leichtle, Johannes Spalinger, Susanne Schibli, Christiane Sokollik

**Affiliations:** 1 University Institute of Clinical Chemistry, Inselspital, Bern University Hospital, University of Bern, Bern, Switzerland; 2 Division of Pediatric Gastroenterology, Hepatology and Nutrition, University Children‘s Hospital, Inselspital, University of Bern, Bern, Switzerland; 3 Division of Pediatric Gastroenterology, Children's Hospital of Lucerne, Lucerne, Switzerland; 4 Division of Pediatric Gastroenterology, Hepatology and Nutrition, Sainte-Justine University Health Centre, Montreal, Canada; 5 IDSC–Insel Data Science Center, Inselspital, Bern University Hospital, University of Bern, Bern, Switzerland; Humanitas University, ITALY

## Abstract

**Background:**

Accurate classification of patients with inflammatory bowel disease into the subtypes ulcerative colitis (UC) and Crohn’s disease (CD) is still a challenge, but important for therapy and prognosis.

**Objectives:**

To evaluate the diagnostic utility of anti-neutrophil cytoplasmic antibodies specific for proteinase-3 (PR3-ANCA) for ulcerative colitis (UC) and the value of an antibody panel incorporating PR3-ANCA to differentiate between Crohn’s disease (CD) and UC.

**Study design:**

In this cohort study, 122 pediatric and adolescent individuals were retrospectively included (61 IBD patients of two clinical centers, 61 non-IBD controls). All subjects had a comprehensive antibody profile done from stored sera taken close to time of diagnosis. By employing quasi-exhaustive logistic regression the best discriminative model for UC and CD,subjects was determined in a training cohort and confirmed in a validation cohort.

**Results:**

PR3-ANCA was specifically associated with UC (odds ratio (OR), 17.6; 95% confidence interval (CI); 3.6, 87); P < .001). A four antibody-panel including PR3-ANCA had an AUC of 90.81% (95%CI; 81.93, 99.69) to distinguish between UC and CD in the training cohort. In a smaller external validation cohort, the AUC was 84.13% (95%CI; 64.21, 100) for accurate diagnosis of CD and UC.

**Conclusion:**

PR3-ANCA is highly specific for UC. The differentiating capability of a panel, which contains PR3-ANCA and weighs broadly available antibodies, is superior and utilization of the panel can support accurate classification in the work-up of pediatric and adolescent patients with IBD patients.

## Introduction

Inflammatory bowel disease (IBD) is a generic term for chronic relapsing inflammatory diseases of the intestine. The main subtypes are Crohn’s disease (CD) and ulcerative colitis (UC). Accurate classification into the subtypes is important for appropriate management. However establishing the correct diagnosis is sometimes challenging due to atypical presentations [1]. Despite a broad work-up based on a combination of history, physical and laboratory examination, esophagogastroduodenoscopy and ileocolonoscopy with histologyas well as imaging of the small bowel in a substantial number of patients a classification is not possible [2] or patients need to be reclassified during the course of the disease [3,4].

The revised Porto criteria for the diagnosis of IBD in children and adolescents discusses the value of antibody testing for diagnosing IBD and concludes that performing antibody testing may help to distinguish between CD and UC [5]. However, the limitation that fewer serological markers are specifically associated with UC than with CD is acknowledged. An important advantage of antibody determination in contrast to other biomarkers such as acute phase proteins, is their relatively stationary prevalence over time regardless of treatment [6–8].

There are two main diagnostic tools for antibody detection, indirect immunofluorescence (IIF), where staining patterns on different tissues and cells can be examined, and chemiluminescent immunoassay (CLIA) as well as enzyme-linked immunosorbent assay (ELISA), which allow a quantitative analysis of antibodies against specific antigens.

Anti-*Saccharomyces cerevisiae* antibodies (ASCA) and anti-neutrophil cytoplasmic antibodies (ANCA) are the most investigated antibodies in IBD. Due to their staining patterns in IIF ANCA can be divided into pANCA with perinuclear, cANCA with cytoplasmic, and xANCA (also called “atypical ANCA”) with a rim-like perinuclear fluorescence (S1 Fig.) [9]. In ANCA-associated vasculitis, pANCA, mainly directed against myeloperoxidase (MPO-ANCA), is a well-established and very specific marker for microscopic polyangiitis, whereas proteinase 3 (PR3)-ANCA is the “classical” cANCA antigen and specific for granulomatosis with polyangiitis also known as Wegener's granulomatosis [10]. Recently, PR3-ANCA not implicit in addition to the “classical” IIF cANCA staining pattern, was shown to be associated with UC in adults [11,12]. PR3-ANCA positivity not only had a comparable sensitivity but also a favorable specificity for UC than classical antibodies or their combinations [11]. Also a prognostic value was suggested as PR3-ANCA reactivity was associated with a more extensive disease location [12].

Other antibodies previously examined for their specificity for a subtype of IBD are exocrine pancreatic autoantibodies (PAB) against glycoproteins of the pancreatic acini for CD [13] and autoantibodies against goblet cells (GAB) for UC [14,15]. Determination of both antibodies may be explicitly important in children and adolescents as PAB antibodies seem to be more prevalent in CD patients diagnosed before the age of 17 years [13]. Reactivity of GAB on gastric tissue (gGAB), suggests that this reactivity is not with goblet cells *per se*, but with components of mucous which can also be found in gastric mucous producing cells [16]. This fact may prime those antibodies to be characteristic of childhood-onset IBD with its higher prevalence of upper gastrointestinal tract disease in comparison to adults [17].

The aim of our study was to investigate the value of PR3-ANCA determination for diagnosing UC in a pediatric and adolescent cohort of IBD patients. Furthermore, we performed an extensive antibody profiling of our cohort to gain an optimal antibody panel to differentiate between CD and UC by employing quasi-exhaustive logistic regression modeling. Therefore, the prevalence of ASCA IgA and IgG, pANCA, cANCA, xANCA, PR3-ANCA, MPO-ANCA, PAB, and gGAB was determined. In a secondary analysis, the capability of antibodies to predict disease phenotype and behavior was assessed.

## Materials and methods

### Study population and design

A total of 61 pediatric and adolescent IBD patients up to the age of 18 years from two centers, the University Children’s Hospital Bern and the Children’s Hospital Lucerne and 61 non-IBD patients were enrolled retrospectively. All UC and CD patients were diagnosed according to the Porto criteria [5,18] and their clinical data was collected by chart review. Clinical data included age at diagnosis and serum sampling, gender, anthropometric measurements, disease specific activity scores (pediatric ulcerative colitis activity index (PUCAI) and weighted pediatric Crohn’s disease activity index (wPCDAI) for UC and CD, respectively), laboratory results at diagnosis and serum sampling, extra-intestinal manifestations (erythema nodosum, arthritis, hepatopathies including primary sclerosing cholangitis and autoimmune hepatitis, uveitis), complications (stricturing or penetrating disease), medical treatment, and need for surgery during follow-up. Disease location at time of diagnosis and disease behavior over time were classified according to the Paris Classification. Age- and sex- matched non-IBD patients were enrolled from our gastroenterology clinics with symptoms resembling IBD such as abdominal pain, diarrhea, rectal bleeding, and weight loss and subsequently diagnosed with functional gastrointestinal disorders according to standard diagnostic criteria [19]. All non-IBD patients had negative serological celiac disease screening. For these patients age and sex at serum sampling were documented. Auto- and antimicrobial antibodies were determined in stored serum samples collected between December 2009 and April 2014. The study was approved by the Ethics Committee of Canton Bern, Switzerland (Ref.-Nr. KEK-BE 018/14) and conducted in accordance with the Helsinki declaration. The ethics committee waived the requirement for written informed consent.

### Analysis of antibodies

All sera were analyzed in a blinded fashion without knowledge of patient diagnosis or other clinical information. For determination of ANCA, sera were incubated on Ethanol-fixed neutrophil granulocytes (INOVA Dx, San Diego). Positive patterns were further divided into cytoplasmic (cANCA), perinuclear (pANCA) or rim-like atypical (xANCA) staining (S1 Fig.). All sera were additionally analyzed for PR3-ANCA and MPO-ANCA using commercial kits based on CLIA Bioflash technology (INOVA Dx, San Diego). ASCA IgA and IgG were determined using commercial ELISA kits (INOVA Dx, San Diego). Antibodies against exocrine pancreas (PAB) antigens rPAg1 (CUZD1) and rPAg2 (GP2) as well as GAB were determined using IIF on cells transfected with the specific antigen (Euroimmun, Lübeck, Germany). gGAB were detected on commercially available murine triple-substrate tissue slides (kidney, stomach, liver; Immco Dx, New York, USA) (S2 Fig.). All tests were performed according to the manufacturer's instructions and were carried out in the routine diagnostic laboratory. Normal reference values as used in routine diagnostic were applied: xANCA, cANCA, pANCA, and gGAB <1:80 titer; PAB and GAB <1:10 titer; ASCA IgA and IgG <20U/ml; PR3-ANCA <5.0U/ml; MPO-ANCA <6.0U/ml.

### Statistical analysis

Univariate analysis was performed using Wilcoxon rank score test for continuous variables, and Chi square or Fisher’s exact test for categorical variables. Sensitivity, specificity, positive and negative predictive value (PPV, NPV) were calculated for each antibody alone or in combination with other antibodies in order to predict the disease subtype.

Based on the Akaike Information Criterion (AIC), an efficient branch-and-bound algorithm for a quasi- exhaustive search for the best subsets of the variables was used [20]. The dichotomous diagnosis class (UC vs CD) was used as dependent and the antibodies as independent variables (R v.3.3.3, package ‘gputools_1.1’) and the model space was assessed using ‘leaps’-based (package ‘leaps_3.0’) wrapper functions. ROC curves were drawn with the pROC package (v. 1.9.1), the computation of optimal cutoffs was done with Youden’s J statistic (package pROC). For power calculations, the power.roc.test() function of the pROC package for R, based on Obuchowski et al was used. [21]. Power calculation revealed that for the training cohort (18 CD and 26 UC patients) the power was 99% using a two-tailed test with alpha set at the conventional level of .05 for the reached AUC of 90.81%. The required sample size for a power of 80% would have been 18 patients. All analyses were performed using SAS software, version 9.3 (SAS Institute, Cary, NC) or R, version 3.3.3 (The R Foundation, Vienna). A p-value <0.05 was considered statistically significant for all analyses.

## Results

### Clinical characteristics

Table 1 shows the clinical characteristics of the study population. Median time lapse between diagnosis of IBD and serum sampling was 4.3 months [interquartile range (IQR): 0, 57.6]. At time of serum sampling most patients had already received treatment at the discretion of the treating physician and activity scores had improved (wPCDAI for CD patients, median [IQR]: 48 [35, 93] at diagnosis vs. 25 [0, 93] at serum sampling; PUCAI for UC patients, median [IQR]: 45 [33, 85] vs. 35 [10, 75], respectively). Laboratory values were not significantly different between UC and CD patients at time of serum sampling (S1 Table). During follow-up, there was no difference in medication use between UC and CD patients apart from the fact, that UC patients received significantly more often mesalazine. Complications included fistulas in 4 (14%) CD patients, strictures in 2 (7%) CD patients, and extraintestinal manifestations in 7 (21%) UC and 10 (36%) CD patients. 1 UC patients and 4 (14%) CD patients needed surgery during follow-up.

**Table 1:** Clinical characteristics of study population.

clinical characteristic	IBD	CD	UC	non-IBD
number of patients	61	28	33	61
males, n (%)	32 (52)	17 (61)	15 (45)	28 (46)
age at diagnosis, median [interquartile range (IQR)], y	11.2 [7.5,12.9]	10.8 [7.4,12.7]	11.3 [8.6,13.4]	-
age at serum sampling, median [IQR], y	13.4 [10.9,15.7]	13.1 [10.8,15.7]	13.8 [11.2,15.7]	12.4 [9.3,14.1]
disease location at diagnosis in CD, n (%)				
L1: ileal		5 (18)		
L2: colonic		8 (29)		
L3: ileocolonic		15 (54)		
L4a: upper disease proximal		13 (46)		
L4b: upper disease distal		3 (11)		
disease location at diagnosis in UC, n (%)				
E1: ulcerative proctitis			0	
E2: left-sided UC (distal to splenic flexure)			6 (18)	
E3: extensive (distal to hepatic flexure)			5 (15)	
E4: pancolitis (proximal to hepatic flexure)			22 (67)	

### Single antibodies and their combinations for diagnosis of ulcerative colitis and Crohn’s disease

The prevalence of antibodies in non-IBD patients was low with 2 (3%) being cANCA, 2 (3%) MPO-ANCA, 2 (3%) ASCA, and 1 (1.5%) gGAB positive. None of the non-IBD controls were positive for PR3-ANCA, pANCA, xANCA, or PAB, resulting in a relatively low sensitivity but high specificity of these antibodies for IBD (S2 Table).

When testing for the differentiating capability of the single antibodies in IBD patients, PR3-ANCA had the most balanced ratio of sensitivity and specificity for UC (58% and 93%, respectively) (Table 2). PAB could be detected in UC as well as CD patients and was not able to distinguish between both disease subtypes in our cohort (OR, 2.1; 95%CI; 0.7, 6.4; P = .208). GAB and gGAB were positive only in a minority of UC and CD patients and it was not possible to differntiate between IBD subtypes.

**Table 2:** Antibody association with ulcerative colitis and Crohn’s disease.

Antibody	CD	UC	p–value	Odds Ratio (95% confidence interval)	Sensitivity, %	Specificity, %	Positive predictive value, %	Positive likelihood ratio
**ANCA**								
cANCA, n (%)	2 (7.4)	6 (18.2)	0.276	2.8 (0.5,15.1)	18	93	75	2.6
PR3-ANCA, n (%)	2 (7.1)	19 (57.6)	<0.001	17.6 (3.6,87)	58	93	90	8.3
PR3-ANCA U/ml, median [IQR]	1 [1, 1.65]	5.8 [1.6,13.8]	<0.001	-	-	-	-	-
pANCA, n (%)	0 (0)	2 (6.1)	0.497	-	6	100	100	-
MPO-ANCA, n (%)	1 (3.6)	3 (9.1)	0.618	2.7 (0.3,27.5)	9	96	75	2.3
MPO-ANCA U/ml, median [IQR]	1 [1,1]	1 [1,1]	0.585	-	-	-	-	-
xANCA, n (%)	4 (14.8)	18 (54.5)	0.003	6.9 (1.9,24.4)	55	93	90	7.9
**GAB**, n (%)	3 (10.7)	1 (3.6)	0.611	3.2 (0.3,33.2)	4	89	25	0.3
**ASCA**								
IgA, n (%)	13 (46.4)	3 (9.1)	0.001	8.7 (2.1,35.1)	46	91	81	5.1
IgA U/ml, median [IQR]	14.5 [7,72.5]	9 [5,13]	0.038	-	-	-	-	-
IgG, n (%)	15 (53.6)	5 (15.2)	0.001	6.5 (1.9,21.6)	54	85	75	3.6
IgG U/ml, median [IQR]	37 [6,70]	7 [4,17]	0.001	-	-	-	-	-
**PAB**, n (%)	10 (35.7)	7 (21.2)	0.208	2.1 (0.7,6.4)	36	79	59	1.7
**gGAB**, n (%)	7 (25)	3 (9.1)	0.164	3.3 (0.8,14.4)	25	91	70	2.8

To improve the diagnostic performance, we combined antibodies in a simple summation. When combining PR3-ANCA positivity and xANCA positivity the specificity for UC increased to 96%, however the sensitivity decreased to 36% with an OR of 15.4 (95%CI; 1.9, 128.3). For PR3-ANCA positivity in combination with ASCA negativity the sensitivity for UC was 52% and the specificity 93% with an OR of 13.8 (95%CI; 2.8, 67.9).

### Diagnostic panel as predictor for ulcerative colitis and Crohn’s disease

**Fig 1:**
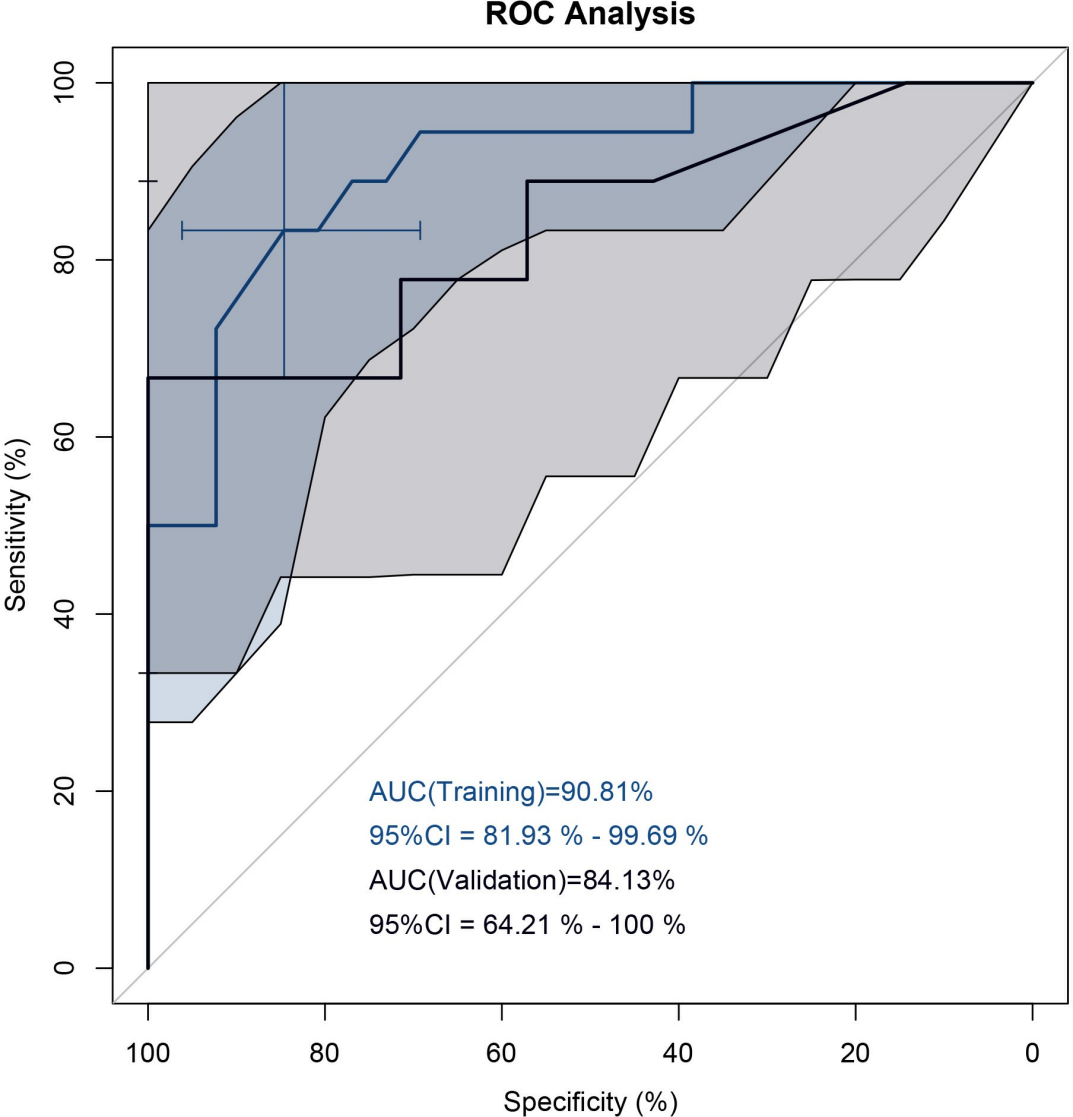
**ROC analysis best fit antibody panel:** Training (blue line) vs validation (black line) cohort. shaded areas represent the 95% confidence interval.

In a next step, the whole antibody profile was used as input for a quasi-exhaustive logistic regression approach to calculate a best-fit antibody combination for prediction of the subtype of IBD, UC and CD. The patients from one center (Bern, n = 44) served as a training cohort (S3 Table). A panel including the positivity status of PR3-ANCA, xANCA, pANCA, and the titer of ASCA IgG was returned. The coefficients for the computation of the predictor in the logistic regression model were as follows: Intercept 0.5937, PR3-ANCA -0.4085, xANCA -0.3280, pANCA -0.6299, and ASCA IgG 0.0052. The best cut-off value for the predictor was 0.6245. The AUC in the training cohort reached 90.81% (95%CI; 81.93, 99.69). The predictive accuracy of the antibody panel could be confirmed in the validation cohort (Lucerne, n = 17) with an AUC of 84.29% (95%CI; 64.75, 100) (Fig 1).

### Antibody profiles and disease behavior

Other than being used as a diagnostic tool, antibody profiling may also predict disease behavior. ASCA IgA positive CD patients had a more complicated and severe disease course (stricturing or penetrating disease, need for surgery) (OR, 12.1; 95%CI; 1.2, 120.1; P = .029). ASCA IgG positive CD patients were more likely to present with ileocolonic disease (L3) (OR, 6.2; 95%CI; 1.2, 32; P = .024). An increased number of positive antibody responses was associated with a more complicated disease course (OR, 12.7; 95%CI; 1.6,120.3; P = .029) in CD patients.

No further associations for different antibodies or their combinations were identified for neither UC nor CD patients. PR3-ANCA positivity was not associated with age at serum sampling. There was no difference in disease extension nor the need for biologicals during follow-up as a marker of disease severity between PR3-ANCA positive and negative UC patients (S4 Table). Of the 2 CD patients with PR3-ANCA positivity one patient had ileocolonic and proximal upper gastrointestinal (A1b, L3, L4a, B1) and the other colonic and proximal upper gastrointestinal (A1a, L2, L4a, B1) disease. There was no significant association (P = .2) between upper disease location (L4a) and gGAB positivity, despite 5/13 (38%) CD patients fulfilling both requirements.

## Discussion

Establishing the diagnosis of IBD and assigning the correct subtype to individual patients can still be challenging. In a pediatric and adolescent cohort of IBD patients, we could show that PR3-ANCA is a strong marker for UC with preeminent sensitivity and specificity. Furthermore, a panel consisting of four antibodies, PR3-ANCA, xANCA, pANCA, and ASCA IgG, showed a very good AUC of 90.8% to distinguish between CD and UC in a training cohort. The validity of this panel was proven in a validation cohort (AUC = 84.1%).

### Antibodies in ulcerative colitis

The presence of ANCA was found to have significant association with UC in our cohort. However, when interpreting and comparing ANCA data between studies varying definitions and determination methods made direct comparison difficult [22]. In some IBD studies the expression “pANCA” has been synonymously used for atypical pANCA, in others there is no differentiation between pANCA and atypical pANCA, and in some studies, the rim-like staining is also referred to as being perinuclear [7,14,23–25]. To be more precise we used the term xANCA only for the rim-like staining pattern as xANCA are the main ANCA found in UC patients when performing IIF [26].

More importantly, none of the non-IBD controls was PR3-ANCA positive and PR3-ANCA positivity had a reasonable sensitivity of 58% and specificity of 93% to distinguish UC from CD patients based on their PR3-ANCA status in our cohort. The positive predictive value for UC was good with 90%. Strikingly, PR3-ANCA were more prevalent in our pediatric and adolescent group with UC (57.6%) than in the published adult groups (31% and 29%)[11,12]. One factor may be that most of our patients already presented with a more extensive disease phenotype. This is consistent with the observation in adults showing a positive association between PR3-ANCA and a more extensive disease location [12,27]. Interestingly, it was also shown in adults that PR3-ANCA reactivity decreases with disease duration, being 48.5% with 0–2 years and 16.7% with 17–20 years [12]. Whether this decrease is due to the effect of treatment can only be answered in longitudinal observations. However, this finding highlights the point that antibody testing should be performed at initial diagnosis.

The combination of PR3-ANCA positivity with ASCA negativity did not improve the sensitivity and specificity for UC in our cohort as it has been described in adults [11]. Yet, combining PR3-ANCA reactivity with xANCA, the specificity for UC increased to 96%. However, the increase in specificity was associated with a simultaneous decrease in sensitivity. This suggested that PR3-ANCA might be of value as a stand-alone marker in the differentiation of UC from CD.

### Antibodies in Crohn’s disease

ASCA is accepted as a highly specific marker for CD [28]. In our cohort ASCA positivity had a reasonable sensitivity of 57% for any ASCA (ASCA IgA and/or ASCA IgG positive) and a specificity of 82%. These numbers are comparable to other published pediatric cohorts with sensitivity of 42% and specificity of 89% for any ASCA [29]. As shown for adults the sensitivity and specificity of ASCA can be optimized, when combining antibody results [30]. When combining ASCA positivity with PR3-ANCA negativity the specificity for CD in our cohort increased to 88%, indicating that a combination with PR3-ANCA reactivity has a higher diagnostic value than focusing on single antibodies.

### Panel diagnostics

The advantage of using a panel over simple summation of single markers is the possibility to weigh each marker and to enhance the granularity. However, most published panels in IBD diagnostic include antibodies that are not available in routine laboratories [7,31–33] or even include genetic in combination with inflammatory markers [31,34]. Additionally, most studies only report on a training cohort and have not included a validation cohort in their analysis.

One pediatric study used ASCA IgA, ASCA IgG, ANCA, and an antibody to the outer membrane protein of *E*. *coli* (OmpC) in a four- antibody panel and achieved a sensitivity of 65% for CD and 76% for UC, with a specificity of 94% [32]. In an adult cohort an antibody panel consisting of ASCA IgA, ASCA IgG, OmpC, anti-flagellin (Cbir1), ANCA, and pANCA discriminated patients with CD and UC with an AUC of 0.78 (95%CI; ± 0.06) [34]. Our panel consisting of ASCA IgG, xANCA, pANCA and PR3-ANCA shows superiority using only four markers, having an AUC in the training cohort of 0.90 (95%CI; 0.81, 0.99) and an AUC of 0.84 (95%CI; 0.64, 1.00) in the external validation cohort. The discrepancy of only 6% of the AUC between the training and validation cohort supports a good validation. The limited number of antibodies also reduced the risk of model over-fitting. Additionally, including only clinically routinely available markers in a panel allows its broad usage. This panel may become a predictor in patients with IBD- unclassified (IBD-U), where the final diagnosis will only be made over time. A longitudinal multicenter study employing our panel is needed to prove this possibility.

### Antibodies for prediction of disease location and behavior

We confirmed in a pediatric population the findings of Elkadri et al. [7] that ASCA positivity in CD patients is associated with ileocolonic disease and can predict a more complicated and severe disease course. Additionally, we also found that an increased number of positive antibody responses is associated with a more complicated disease pattern in CD patients [7].

Recently the interest in PAB as a marker for CD has increased [35]. However, in comparison to adult studies PAB reactivity was not able to distinguish between CD and UC in our cohort [13,24]. The high prevalence of PAB reactivity in our UC patients, which was also found in another pediatric study, may explain this finding [14]. In concordance with the findings of Kovacs [14], we could not demonstrate an association between PAB positivity and extra-intestinal manifestations, complicated disease course or need for therapy with biologics in pediatric patients as suggested in adults [36].

Although GAB is thought to be associated with UC, we found it more often in CD patients. However, in total only 7% IBD patients in our cohort were positive, which is a much lower prevalence than previously described in adults being around 30% [37]. In an IIF study using human tissue from different intestinal locations a trend was seen with CD sera preferentially staining goblet cells in the small intestine and UC sera staining goblet cells in the sigmoid and rectum [16]. This may suggest a possible relation between reactivity pattern and disease location. In our study however, there was no association between upper GI tract disease and gGAB reactivity when using murine gastric tissue. Further studies including more patients with upper GI tract disease location are needed to draw a conclusion of the value of gGAB determination.

### Strengths and limitations

We acknowledge the small sample size as the main limitation of our study. However, including patients with a short time lapse from diagnosis and serum sampling allows concluding that PR3-ANCA determination can be of value in early differentiation of UC from CD patients based on their serological antibody profile. Another strength is the employment of patients from two different centers. This allowed us to use the proposed antibody panel in a training and in a validation cohort, which improved the validity of the panel. Additionally, the use of routinely available markers makes the information accessible and useful for the clinician.

### Conclusion

PR3-ANCA and the combination of antibodies in a panel consisting of PR3-ANCA, pANCA, xANCA, and ASCA IgG improves diagnostic accuracy in differentiating between CD and UC. The prognostic value of antibody determination however seems to be limited as prediction of disease behavior was indefinite when compared to published associations.

## Supporting information

S1 Fig Different immunofluorescent patterns of ANCA. A) cANCA with cytoplasmic B) pANCA with perinuclear, and C) (atypical) xANCA with a rim-like perinuclear fluorescence.
(TIF)

S2 Fig Gastric GAB staining on commercial triple-substrate tissue slide. Kidney (■), smooth muscle (*), stomach (●). Kidney is surrounded by stomach tissue including the smooth muscle rim. Liver tissue not visible on this part of the slide.
(TIF)

S1 Table Laboratory values in IBD patients. (DOCX)

S2 Table Antibodies in non-IBD vs IBD patients. (DOCX)

S3 Table Characteristics and antibody status of the training and validation cohort. (DOCX)

S4 Table PR3-ANCA status and disease behavior in UC patients. (DOCX)

## Abbreviations

PR3-ANCA: proteinase-3 anti-neutrophil cytoplasmic antibodies
MPO-ANCA: myeloperoxidase anti-neutrophil cytoplasmic antibodies
ASCA: anti- *Saccharomyces cerevisiae* antibodies
xANCA: atypical anti-neutrophil cytoplasmic antibodies
GAB: autoantibodies against goblet cells
CD: Crohn’s disease
cANCA: cytoplasmic anti-neutrophil cytoplasmic antibodies
PAB: exocrine pancreatic autoantibodies
gGAB: gastric autoantibodies against goblet cells
IBD: inflammatory bowel disease
pANCA: perinuclear anti-neutrophil cytoplasmic antibodies
UC: ulcerative colitis

## References

[1] Levine A, de Bie CI, Turner D, Cucchiara S, Sladek M, Murphy MS, et al (2013) Atypical disease phenotypes in pediatric ulcerative colitis: 5-year analyses of the EUROKIDS Registry. Inflamm Bowel Dis 19: 370–377. 10.1002/ibd.23013 22570259 10.1002/ibd.23013

[2] Carvalho RS, Abadom V, Dilworth HP, Thompson R, Oliva-Hemker M, Cuffari C (2006) Indeterminate colitis: a significant subgroup of pediatric IBD. Inflamm Bowel Dis 12: 258–262. 10.1097/01.MIB.0000215093.62245.b9 16633047 10.1097/01.MIB.0000215093.62245.b9

[3] Matsui T, Yao T, Sakurai T, Yao K, Hirai F, Matake H, et al (2003) Clinical features and pattern of indeterminate colitis: Crohn's disease with ulcerative colitis-like clinical presentation. J Gastroenterol 38: 647–655. 10.1007/s00535-003-1117-8 12898357 10.1007/s00535-003-1117-8

[4] Alexander F, Sarigol S, DiFiore J, Stallion A, Cotman K, Clark H, et al (2003) Fate of the pouch in 151 pediatric patients after ileal pouch anal anastomosis. J Pediatr Surg 38: 78–82. 10.1053/jpsu.2003.50015 12592624 10.1053/jpsu.2003.50015

[5] Levine A, Koletzko S, Turner D, Escher JC, Cucchiara S, de Ridder L, et al (2014) ESPGHAN revised porto criteria for the diagnosis of inflammatory bowel disease in children and adolescents. J Pediatr Gastroenterol Nutr 58: 795–806. 10.1097/MPG.0000000000000239 24231644 10.1097/MPG.0000000000000239

[6] Olbjorn C, Cvancarova Smastuen M, Thiis-Evensen E, Nakstad B, Vatn MH, Perminow G (2017) Serological markers in diagnosis of pediatric inflammatory bowel disease and as predictors for early tumor necrosis factor blocker therapy. Scand J Gastroenterol 52: 414–419. 10.1080/00365521.2016.1259653 27887202 10.1080/00365521.2016.1259653

[7] Elkadri AA, Stempak JM, Walters TD, Lal S, Griffiths AM, Steinhart AH, et al (2013) Serum antibodies associated with complex inflammatory bowel disease. Inflamm Bowel Dis 19: 1499–1505. 10.1097/MIB.0b013e318281f2a1 23702714 10.1097/MIB.0b013e318281f2a1

[8] Teml A, Kratzer V, Schneider B, Lochs H, Norman GL, Gangl A, et al (2003) Anti-Saccharomyces cerevisiae antibodies: a stable marker for Crohn's disease during steroid and 5-aminosalicylic acid treatment. Am J Gastroenterol 98: 2226–2231. 10.1111/j.1572-0241.2003.07673.x 14572572 10.1111/j.1572-0241.2003.07673.x

[9] Gross WL, Schmitt WH, Csernok E (1993) ANCA and associated diseases: immunodiagnostic and pathogenetic aspects. Clin Exp Immunol 91: 1–12. 8419069 10.1111/j.1365-2249.1993.tb03345.x PMC1554662

[10] Bossuyt X, Cohen Tervaert JW, Arimura Y, Blockmans D, Flores-Suarez LF, Guillevin L, et al (2017) Position paper: Revised 2017 international consensus on testing of ANCAs in granulomatosis with polyangiitis and microscopic polyangiitis. Nat Rev Rheumatol 13: 683–692. 10.1038/nrrheum.2017.140 28905856 10.1038/nrrheum.2017.140

[11] Arias-Loste MT, Bonilla G, Moraleja I, Mahler M, Mieses MA, Castro B, et al (2013) Presence of anti-proteinase 3 antineutrophil cytoplasmic antibodies (anti-PR3 ANCA) as serologic markers in inflammatory bowel disease. Clin Rev Allergy Immunol 45: 109–116. 10.1007/s12016-012-8349-4 23345025 10.1007/s12016-012-8349-4

[12] Mahler M, Bogdanos DP, Pavlidis P, Fritzler MJ, Csernok E, Damoiseaux J, et al (2013) PR3-ANCA: a promising biomarker for ulcerative colitis with extensive disease. Clin Chim Acta 424: 267–273. 10.1016/j.cca.2013.06.005 23806819 10.1016/j.cca.2013.06.005

[13] Bogdanos DP, Roggenbuck D, Reinhold D, Wex T, Pavlidis P, von Arnim U, et al (2012) Pancreatic-specific autoantibodies to glycoprotein 2 mirror disease location and behaviour in younger patients with Crohn's disease. BMC Gastroenterol 12: 102 10.1186/1471-230X-12-102 22866900 10.1186/1471-230X-12-102 PMC3449192

[14] Kovacs M, Lakatos PL, Papp M, Jacobsen S, Nemes E, Polgar M, et al (2012) Pancreatic autoantibodies and autoantibodies against goblet cells in pediatric patients with inflammatory bowel disease. J Pediatr Gastroenterol Nutr 55: 429–435. 10.1097/MPG.0b013e318256b516 22465933 10.1097/MPG.0b013e318256b516

[15] Homsak E, Micetic-Turk D, Bozic B (2010) Autoantibodies pANCA, GAB and PAB in inflammatory bowel disease: prevalence, characteristics and diagnostic value. Wien Klin Wochenschr 122 Suppl 2: 19–25. 10.1007/s00508-010-1344-y 20517666

[16] Ardesjo B, Portela-Gomes GM, Rorsman F, Gerdin E, Loof L, Grimelius L, et al (2008) Immunoreactivity against Goblet cells in patients with inflammatory bowel disease. Inflamm Bowel Dis 14: 652–661. 10.1002/ibd.20370 18213698 10.1002/ibd.20370

[17] Van Limbergen J, Russell RK, Drummond HE, Aldhous MC, Round NK, Nimmo ER, et al (2008) Definition of phenotypic characteristics of childhood-onset inflammatory bowel disease. Gastroenterology 135: 1114–1122. 10.1053/j.gastro.2008.06.081 18725221 10.1053/j.gastro.2008.06.081

[18] (2005) Inflammatory bowel disease in children and adolescents: recommendations for diagnosis—the Porto criteria. J Pediatr Gastroenterol Nutr 41: 1–7. 15990620 10.1097/01.mpg.0000163736.30261.82

[19] Rasquin A, Di Lorenzo C, Forbes D, Guiraldes E, Hyams JS, Staiano A, et al (2006) Childhood functional gastrointestinal disorders: child/adolescent. Gastroenterology 130: 1527–1537. 10.1053/j.gastro.2005.08.063 16678566 10.1053/j.gastro.2005.08.063 PMC7104693

[20] Miller AJ (1984) Selection of Subsets of Regression Variables Journal of the Royal Statistical Society 147: 36.

[21] Obuchowski NA, Lieber ML, Wians FH Jr. (2004) ROC curves in clinical chemistry: uses, misuses, and possible solutions. Clin Chem 50: 1118–1125. 10.1373/clinchem.2004.031823 15142978 10.1373/clinchem.2004.031823

[22] Jaskowski TD, Litwin CM, Hill HR (2006) Analysis of serum antibodies in patients suspected of having inflammatory bowel disease. Clin Vaccine Immunol 13: 655–660. 10.1128/CVI.00034-06 16760323 10.1128/CVI.00034-06 PMC1489548

[23] Oudkerk Pool M, Ellerbroek PM, Ridwan BU, Goldschmeding R, von Blomberg BM, Pena AS, et al (1993) Serum antineutrophil cytoplasmic autoantibodies in inflammatory bowel disease are mainly associated with ulcerative colitis. A correlation study between perinuclear antineutrophil cytoplasmic autoantibodies and clinical parameters, medical, and surgical treatment. Gut 34: 46–50. 8432451 10.1136/gut.34.1.46 PMC1374099

[24] Michaels MA, Jendrek ST, Korf T, Nitzsche T, Teegen B, Komorowski L, et al (2015) Pancreatic Autoantibodies Against CUZD1 and GP2 Are Associated with Distinct Clinical Phenotypes of Crohn's Disease. Inflamm Bowel Dis 21: 2864–2872. 10.1097/MIB.0000000000000564 26273818 10.1097/MIB.0000000000000564

[25] Mow WS, Vasiliauskas EA, Lin YC, Fleshner PR, Papadakis KA, Taylor KD, et al (2004) Association of antibody responses to microbial antigens and complications of small bowel Crohn's disease. Gastroenterology 126: 414–424. 14762777 10.1053/j.gastro.2003.11.015

[26] Frenzer A, Fierz W, Rundler E, Hammer B, Binek J (1998) Atypical, cytoplasmic and perinuclear anti-neutrophil cytoplasmic antibodies in patients with inflammatory bowel disease. J Gastroenterol Hepatol 13: 950–954. 9794196 10.1111/j.1440-1746.1998.tb00767.x

[27] Takedatsu H, Mitsuyama K, Fukunaga S, Yoshioka S, Yamauchi R, Mori A, et al (2018) Diagnostic and clinical role of serum proteinase 3 antineutrophil cytoplasmic antibodies in inflammatory bowel disease. J Gastroenterol Hepatol. 10.1111/jgh.14140 29514409

[28] Reese GE, Constantinides VA, Simillis C, Darzi AW, Orchard TR, Fazio VW, et al (2006) Diagnostic precision of anti-Saccharomyces cerevisiae antibodies and perinuclear antineutrophil cytoplasmic antibodies in inflammatory bowel disease. Am J Gastroenterol 101: 2410–2422. 10.1111/j.1572-0241.2006.00840.x 16952282 10.1111/j.1572-0241.2006.00840.x

[29] Russell RK, Ip B, Aldhous MC, MacDougall M, Drummond HE, Arnott ID, et al (2009) Anti-Saccharomyces cerevisiae antibodies status is associated with oral involvement and disease severity in Crohn disease. J Pediatr Gastroenterol Nutr 48: 161–167. 10.1097/MPG.0b013e318183e112 19179877 10.1097/MPG.0b013e318183e112

[30] Peeters M, Joossens S, Vermeire S, Vlietinck R, Bossuyt X, Rutgeerts P (2001) Diagnostic value of anti-Saccharomyces cerevisiae and antineutrophil cytoplasmic autoantibodies in inflammatory bowel disease. Am J Gastroenterol 96: 730–734. 10.1111/j.1572-0241.2001.03613.x 11280542 10.1111/j.1572-0241.2001.03613.x

[31] Schoepfer AM, Schaffer T, Mueller S, Flogerzi B, Vassella E, Seibold-Schmid B, et al (2009) Phenotypic associations of Crohn's disease with antibodies to flagellins A4-Fla2 and Fla-X, ASCA, p-ANCA, PAB, and NOD2 mutations in a Swiss Cohort. Inflamm Bowel Dis 15: 1358–1367. 10.1002/ibd.20892 19253375 10.1002/ibd.20892 PMC2732763

[32] Zholudev A, Zurakowski D, Young W, Leichtner A, Bousvaros A (2004) Serologic testing with ANCA, ASCA, and anti-OmpC in children and young adults with Crohn's disease and ulcerative colitis: diagnostic value and correlation with disease phenotype. Am J Gastroenterol 99: 2235–2241. 10.1111/j.1572-0241.2004.40369.x 15555007 10.1111/j.1572-0241.2004.40369.x

[33] Paul S, Boschetti G, Rinaudo-Gaujous M, Moreau A, Del Tedesco E, Bonneau J, et al (2015) Association of Anti-glycan Antibodies and Inflammatory Bowel Disease Course. J Crohns Colitis 9: 445–451. 10.1093/ecco-jcc/jjv063 25895876 10.1093/ecco-jcc/jjv063

[34] Plevy S, Silverberg MS, Lockton S, Stockfisch T, Croner L, Stachelski J, et al (2013) Combined serological, genetic, and inflammatory markers differentiate non-IBD, Crohn's disease, and ulcerative colitis patients. Inflamm Bowel Dis 19: 1139–1148. 10.1097/MIB.0b013e318280b19e 23518807 10.1097/MIB.0b013e318280b19e PMC3792797

[35] Komorowski L, Teegen B, Probst C, Aulinger-Stocker K, Sina C, Fellermann K, et al (2013) Autoantibodies against exocrine pancreas in Crohn's disease are directed against two antigens: the glycoproteins CUZD1 and GP2. J Crohns Colitis 7: 780–790. 10.1016/j.crohns.2012.10.011 23140841 10.1016/j.crohns.2012.10.011

[36] Papp M, Sipeki N, Tornai T, Altorjay I, Norman GL, Shums Z, et al (2015) Rediscovery of the Anti-Pancreatic Antibodies and Evaluation of their Prognostic Value in a Prospective Clinical Cohort of Crohn's Patients: The Importance of Specific Target Antigens [GP2 and CUZD1]. J Crohns Colitis 9: 659–668. 10.1093/ecco-jcc/jjv087 25968583 10.1093/ecco-jcc/jjv087

[37] Hibi T, Ohara M, Kobayashi K, Brown WR, Toda K, Takaishi H, et al (1994) Enzyme linked immunosorbent assay (ELISA) and immunoprecipitation studies on anti-goblet cell antibody using a mucin producing cell line in patients with inflammatory bowel disease. Gut 35: 224–230. 8307474 10.1136/gut.35.2.224 PMC1374498

