# Supplementary figures and images for: PR3-ANCA and panel diagnostics in pediatric inflammatory bowel disease to distinguish ulcerative colitis from Crohn's disease
Source: PLoS One. 2018 Dec 17;13(12):e0208974. doi: 10.1371/journal.pone.0208974 (PMC6296712; doi:10.1371/journal.pone.0208974)

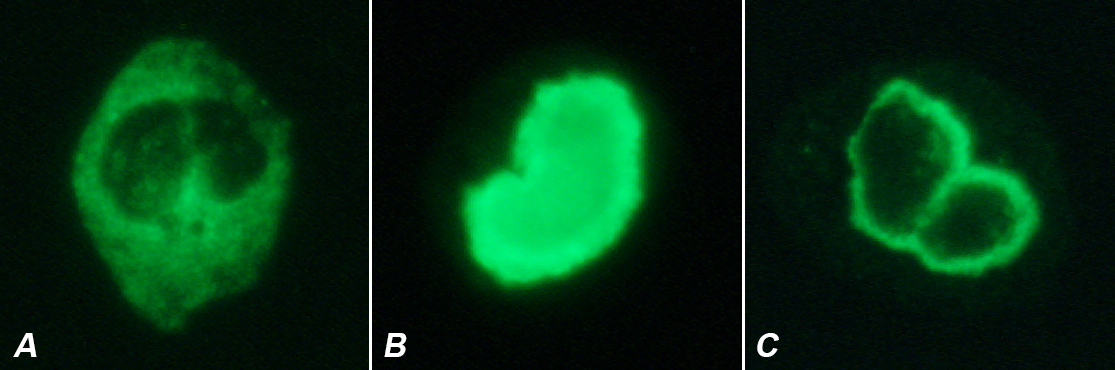

Supplement: S1 Fig — A) cANCA with cytoplasmic B) pANCA with perinuclear, and C) (atypical) xANCA with a rim-like perinuclear fluorescence. (TIF) [file pone.0208974.s001.tif]

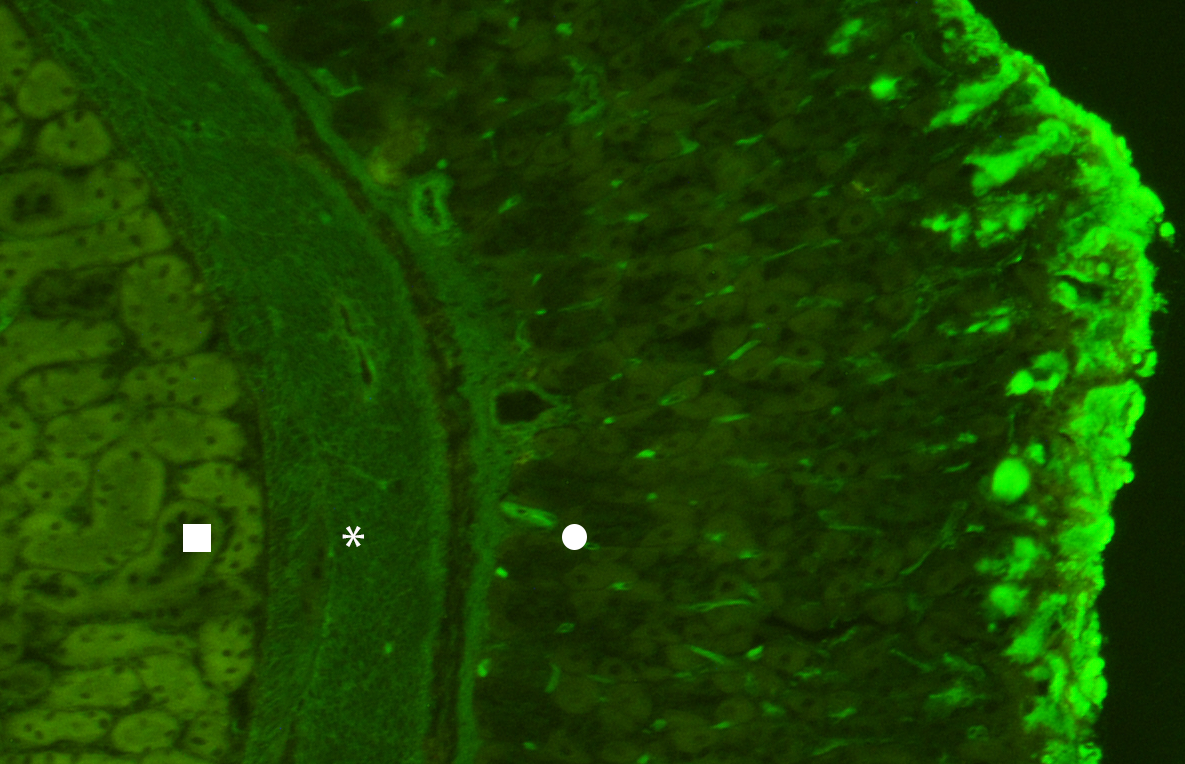

Supplement: S2 Fig — Kidney (■), smooth muscle (*), stomach (●). Kidney is surrounded by stomach tissue including the smooth muscle rim. Liver tissue not visible on this part of the slide. (TIF) [file pone.0208974.s002.tif]
