# Supplementary material for: PR3-ANCA and panel diagnostics in pediatric inflammatory bowel disease to distinguish ulcerative colitis from Crohn's disease
Source: PLoS One. 2018 Dec 17;13(12):e0208974. doi: 10.1371/journal.pone.0208974 (PMC6296712; doi:10.1371/journal.pone.0208974)
Supplement: S1 Table — (DOCX) [file pone.0208974.s003.docx]

**S1 Table.** Laboratory values in IBD patients.

| **Laboratory work-up** | **CD** | **UC** | **p – value** |
| --- | --- | --- | --- |
|  | n=28 | n=33 | CD vs. UC |
| at time of diagnosis, median [interquartile range] |  |  |  |
| Hemoglobine, g/dl, CD=23, UC=31 | 11.3 [9.6, 11.8] | 11.0 [9.3, 12.4] | 0.746 |
| Thrombocytes, x10^3^/μL, CD=22, UC=32 | 476 [383, 554] | 403 [342, 495] | 0.205 |
| Leucocytes /L, CD=22, UC=32 | 9400 [7800, 10100] | 9400[6900, 11000] | 0.526 |
| Albumine g/dL, CD=20, UC=26 | 3.2 [2.8, 3.7] | 3.6 [3.1, 3.9] | 0.398 |
| C-reactive protein mg/L, CD=22, UC=28 | 1.7 [0.8, 5.1] | 1.8 [0.3, 2.2] | 0.072 |
| Erythrocyte sedimentation rate, mm/hr, CD=18, UC=20 | 45 [17, 60] | 28 [21, 45] | 0.306 |
| Fecal calprotectin, g/kg, CD=10, UC=18 | 570 [473, 623] | 698 [600, 3000] | 0.076 |
| at time of serum sampling, median [interquartile range] |  |  |  |
| Hemoglobine, g/dL, CD=28, UC=33 | 12.1 [11.5, 13.5] | 11.7 [11.0, 13.3] | 0.409 |
| Thrombocytes, x10^3^/μL, CD=28, UC=33 | 354 [269, 450] | 360 [314, 469] | 0.439 |
| Leucocytes /L, CD=28, UC=33 | 8100 [6700, 9300] | 8200 [7000, 9500] | 0.422 |
| Albumine g/dl, CD=28, UC=31 | 38 [31, 42] | 37 [33, 40] | 0.616 |
| C-reactive protein mg/L, CD=28, UC=32 | 0.5 [0.3, 1.8] | 0.5 [0.3, 1.6] | 0.539 |
| Erythrocyte sedimentation rate, mm/hr, CD=24, UC=26 | 21 [8, 54] | 18 [9, 33] | 0.611 |
| Fecal calprotectin, g/kg, CD=24, UC=21 | 515 [94, 688] | 605 [200, 785] | 0.169 |
