# Supplementary material for: PR3-ANCA and panel diagnostics in pediatric inflammatory bowel disease to distinguish ulcerative colitis from Crohn's disease
Source: PLoS One. 2018 Dec 17;13(12):e0208974. doi: 10.1371/journal.pone.0208974 (PMC6296712; doi:10.1371/journal.pone.0208974)
Supplement: S2 Table — (DOCX) [file pone.0208974.s004.docx]

**S2 Table.** Antibodies in non-IBD vs IBD patients.

| Antibody | non-IBD | IBD | p – value | Odds ratio, 95% Confidence interval | Sensitivity | Specificity | positive predictive value | positive likelihood ratio |
| --- | --- | --- | --- | --- | --- | --- | --- | --- |
|  | n=61 | n=61 | IBD vs. non-IBD | IBD vs. non-IBD | % | % | % |  |
| ANCA |  |  |  |  |  |  |  |  |
| cANCA, n (%) | 2 (3) | 8 (13) | 0.054 | 4.5 (0.9, 22.3) | 13 | 97 | 80 | 4.3 |
| PR3-ANCA, n (%) | 0 (0) | 21 (34) | <.001 | - | 34 | 100 | 100 | - |
| pANCA, n (%) | 0 (0) | 2 (3) | 0.244 | - | 3 | 100 | 100 | - |
| MPO-ANCA, n (%) | 2 (3) | 4 (7) | 0.680 | 2.1 (0.4, 11.7) | 7 | 97 | 67 | 2.3 |
| xANCA, n (%) | 0 (0) | 22 (37) | <0.001 | - | 37 | 100 | 100 | - |
| ASCA |  |  |  |  |  |  |  |  |
| IgA, n (%) | 2 (3) | 16 (26) | <.001 | 10.5 (2.3, 47.9) | 26 | 97 | 89 | 8.7 |
| IgG, n (%) | 1 (2) | 20 (33) | <.001 | 29.3 (3.8, 226.7) | 33 | 98 | 95 | 16.5 |
| IgA / IgG, n (%) | 2 (3) | 22 (36) | <.001 | 16.6 (3.7, 74.8) | 36 | 97 | 92 | 12 |
| Gastric GAB (gGAB), n (%) | 1 (2) | 10 (16) | 0.004 | 11.8 (1.5, 95.0) | 16 | 98 | 91 | 8 |
