# Supplementary material for: PR3-ANCA and panel diagnostics in pediatric inflammatory bowel disease to distinguish ulcerative colitis from Crohn's disease
Source: PLoS One. 2018 Dec 17;13(12):e0208974. doi: 10.1371/journal.pone.0208974 (PMC6296712; doi:10.1371/journal.pone.0208974)
Supplement: S3 Table — (DOCX) [file pone.0208974.s005.docx]

**S3 Table.** Clinical characteristics and antibody status of the training and validation cohort.

| **Characteristic** | **Training cohort** (Bern) | | **Validation cohort** (Lucerne) | |
| --- | --- | --- | --- | --- |
|  | **CD** | **UC** | **CD** | **UC** |
| number of patients | 18 | 26 | 10 | 7 |
| age at diagnosis, median [IQR], y | 8.3 [7.2,12.6] | 11.2 [7.4,12.6] | 11.7 [8.3,12.9] | 11.9 [9.2,13.9] |
| age at serum sampling, median [IQR], y | 12.7 [10.7,14.7] | 13.2 [10.8,15.7] | 13.9 [11.9,16.6] | 13.8 [11.2,15.8] |
| disease location at diagnosis in CD, n (%) |  |  |  |  |
| L1: ileal | 3 (17) |  | 2 (20) |  |
| L2: colonic | 6 (33) |  | 2 (20) |  |
| L3: ileocolonic | 9 (50) |  | 6 (60) |  |
| L4a: upper disease proximal | 7 (39) |  | 6 (60) |  |
| L4b: upper disease distal | 2 (11) |  | 1 (10) |  |
| disease location at diagnosis in UC, n (%) |  |  |  |  |
| E1: ulcerative proctitis |  | 0 (0) |  | 0 (0) |
| E2: left-sided UC (distal to splenic flexure) |  | 5 (19) |  | 1 (14) |
| E3: extensive (distal to hepatic flexure) |  | 4 (15) |  | 1 (14) |
| E4: pancolitis (proximal to hepatic flexure) |  | 17 (65) |  | 5 (71) |
| **Antibodies** |  |  |  |  |
| PR3-ANCA, n (%) | 1 (6) | 6 (23) | 1 (10) | 4 (57) |
| xANCA, n (%) | 2 (11) | 15 (58) | 2 (20) | 3 (43) |
| pANCA, n (%) | 0 (0) | 1 (4) | 0 (0) | 1 (14) |
| ASCA IgG U/ml, median [IQR] | 17.5 [6,63] | 6.5 [4,14] | 47.5 [7,94] | 9 [4,22] |
