# Supplementary material for: PR3-ANCA and panel diagnostics in pediatric inflammatory bowel disease to distinguish ulcerative colitis from Crohn's disease
Source: PLoS One. 2018 Dec 17;13(12):e0208974. doi: 10.1371/journal.pone.0208974 (PMC6296712; doi:10.1371/journal.pone.0208974)
Supplement: S4 Table — (DOCX) [file pone.0208974.s006.docx]

**S4 Table.** PR3-ANCA status and disease behavior in UC patients.

|  | PR3- ANCA positive  (n = 19) | PR3-ANCA negative  (n = 14) | p-value |
| --- | --- | --- | --- |
| Age at diagnosis, median [IQR] | 11.5 [10.7,13.6] | 9.2 [ 4.9,11.9] | 0.05 |
| **Paris Classification of initial disease in UC, n (%)** |  |  |  |
| E1: ulcerative proctitis | 0 | 0 |  |
| E2: Left-sided UC (distal to splenic flexure) | 5 (26) | 1 (7) | 0.21 |
| E3: Extensive (distal to hepatic flexure) | 3 (16) | 2 (14) | 1.0 |
| E4: Pancolitis (proximal to hepatic flexure) | 11 (58) | 11 (79) | 0.28 |
| **PUCAI, median [IQR]** |  |  |  |
| at time of diagnosis | 47.5 [35.0, 57.5] | 45 [20.0, 60.0] | 0.83 |
| at time of serum sampling | 35.0 [30.0, 50.0] | 15.0 [0,35.0] | 0.04 |
| **Disease course, n (%)** |  |  |  |
| Need for surgery | 1 (5) | 0 | 1.00 |
| Extraintestinal manifestation | 5 (26) | 2 (14) | 0.67 |
| **Therapy, n (%)** |  |  |  |
| Biologics (anti-TNF-α antibodies) | 6 (32) | 5 (36) | 1.00 |
